# Transcriptome Analysis During Follicle Development in Turkey Hens With Low and High Egg Production

**DOI:** 10.3389/fgene.2021.619196

**Published:** 2021-03-18

**Authors:** Kristen Brady, Hsiao-Ching Liu, Julie A. Hicks, Julie A. Long, Tom E. Porter

**Affiliations:** ^1^Department of Animal and Avian Sciences, University of Maryland, College Park, MD, United States; ^2^Animal Biosciences and Biotechnology Laboratory, Beltsville Agricultural Research Center (BARC), Agricultural Research Service (ARS), United States Department of Agriculture (USDA), Beltsville, MD, United States; ^3^Department of Animal Science, North Carolina State University, Raleigh, NC, United States

**Keywords:** turkey, RNA-seq, thyroid hormone, estradiol, HPT axis, HPG axis, steroidogenesis, egg production

## Abstract

Low and high egg producing hens exhibit gene expression differences related to ovarian steroidogenesis. High egg producing hens display increased expression of genes involved in progesterone and estradiol production, in the granulosa layer of the largest follicle (F1G) and small white follicles (SWF), respectively, whereas low egg producing hens display increased expression of genes related to progesterone and androgen production in the granulosa (F5G) and theca interna layer (F5I) of the fifth largest follicle, respectively. Transcriptome analysis was performed on F1G, F5G, F5I, and SWF samples from low and high egg producing hens to identify novel regulators of ovarian steroidogenesis. In total, 12,221 differentially expressed genes (DEGs) were identified between low and high egg producing hens across the four cell types examined. Pathway analysis implied differential regulation of the hypothalamo-pituitary-thyroid (HPT) axis, particularly thyroid hormone transporters and thyroid hormone receptors, and of estradiol signaling in low and high egg producing hens. The HPT axis showed up-regulation in high egg producing hens in less mature follicles but up-regulation in low egg producing hens in more mature follicles. Estradiol signaling exclusively exhibited up-regulation in high egg producing hens. Treatment of SWF cells from low and high egg producing hens with thyroid hormone *in vitro* decreased estradiol production in cells from high egg producing hens to the levels seen in cells from low egg producing hens, whereas thyroid hormone treatment did not impact estradiol production in cells from low egg producing hens. Transcriptome analysis of the major cell types involved in steroidogenesis inferred the involvement of the HPT axis and estradiol signaling in the regulation of differential steroid hormone production seen among hens with different egg production levels.

## Introduction

Avian species display an ovarian hierarchy, with follicles in all stages of development present in the ovary at any given time during egg production (Johnson, 2015a). Four types of follicles exist during follicle development: primordial follicles, primary follicles, prehierarchical follicles, and preovulatory follicles. Maintenance of the follicular hierarchy is achieved by coordinated activation of primordial follicles to grow into primary follicles, recruitment of primary follicles to develop into prehierarchical follicles, selection of prehierarchical follicles to join the cohort of preovulatory follicles, and ovulation of preovulatory follicles to undergo egg formation in the oviduct (Johnson, 2014). The prehierarchical follicles and preovulatory follicles are responsible for the majority of ovarian steroid hormone production. Throughout follicle maturation, steroid production shifts from estradiol production in less mature follicles, to androgen production in follicles in the middle stages of development, then to progesterone production in more mature follicles (Porter et al., 1991). Along with this shift, a change in gonadotropin sensitivity is seen as well, shifting from follicle stimulating hormone (FSH) responsiveness in less mature follicles to luteinizing hormone (LH) responsiveness in more mature follicles (Johnson and Woods, 2009).

Ovarian steroidogenesis in birds occurs through a three-cell model, in which the three cell types of the follicle wall, the granulosa cells, the theca interna cells, and the theca externa cells, produce progesterone, androgens, and estradiol, respectively (Porter et al., 1989). Small white follicles (SWF), a type of prehierarchical follicle, produce the majority of estradiol in the ovary (Johnson, 1992). The theca interna layer of the fifth largest follicle (F5I), which is one of the preovulatory follicles, produces the majority of androgens in the ovary (Porter et al., 1991). The granulosa layer of the largest follicle (F1G), which is the next preovulatory follicle to ovulate, produces the majority of progesterone in the ovary (Bahr et al., 1983).

Steroid hormones produced in the ovary feedback on the hypothalamus and pituitary to regulate the activity of the hypothalamo-pituitary-gonadal (HPG) axis (Johnson et al., 1985; Li et al., 1994). The HPG axis is the main regulator of ovulation rates in avian and mammalian species (Paster, 1991). Among commercial turkey breeding hens, variation in ovulation rates are observed within a single flock (Brady et al., 2020a). Distinct groups of low and high egg producing hens are observed in the top and bottom 15% of the flock. Low egg producing hens significantly impact the number of poults produced that can be raised for meat production and cost more to maintain per egg laid than high egg producing hens. Despite differences in ovulation rates, macroscopic morphology of the ovary does not differ between low and high egg producing hens, indicating that differences in the function of the HPG axis may be responsible for differential egg production levels (Brady et al., 2020a). Targeted gene expression approaches in low and high egg producing hens revealed differential expression of steroidogenic genes in the ovary (Brady et al., 2020a). Low egg producing hens showed up-regulation of genes consistent with increased androgen production in the F5I relative to high egg producing hens. High egg producing hens showed up-regulation of genes consistent with increased progesterone and estradiol production in the F1G and SWF, respectively, relative to low egg producing hens. Interestingly, low egg producing hens also showed up-regulation of genes consistent with increased progesterone production in the granulosa layer of the fifth largest preovulatory follicle (F5G) compared to high egg producing hens, suggesting that movement through the follicular hierarchy may occur at a slower rate in low egg producing hens. Increased progesterone and estradiol production in the F1G and SWF cells from high egg producing hens was also seen *in vitro* following LH and FSH treatment (Brady et al., 2020b).

Both steroid hormone production and follicle development are influenced by the hypothalamo-pituitary-thyroid (HPT) axis and by paracrine effects of steroid hormones in the ovary (Rangel et al., 2009; Sechman, 2012). Steroid hormone and thyroid hormone receptors are expressed in ovarian follicles at each stage of development (Sechman et al., 2009; González-Morán et al., 2013). Furthermore, *in vitro* steroid hormone and thyroid hormone treatment impacts steroidogenesis, with estradiol generally increasing steroidogenesis and thyroid hormone generally decreasing steroidogenesis (Sechman et al., 2009; Caicedo Rivas et al., 2016). Transcriptome analysis was performed in the F1G, F5G, F5I, and SWF of low and high egg producing hens to obtain a more global understanding of the regulation of steroid hormone production and follicle development in low and high egg producing hens.

## Results

### Transcriptome Alignment and Mapping

A total of 788,763,171 sequence reads were obtained from the four follicle cell types examined, with an average of 32,865,132 reads per sample (Supplementary Figure S1A). On average, 77.1% of reads mapped to the turkey reference genome (Turkey_2.01). For each sample, read pairs were aligned with minimal discordant pairs or pairs with multiple alignments (average of 0.82 and 3.84%, respectively) (Supplementary Figure S1B). The number of reads per sample, the number of mapped reads per sample, and the number of properly aligned pairs per sample did not differ significantly between low and high egg producing hens in any of the tissues examined.

### Overview of DEGs

**FIGURE 1:**
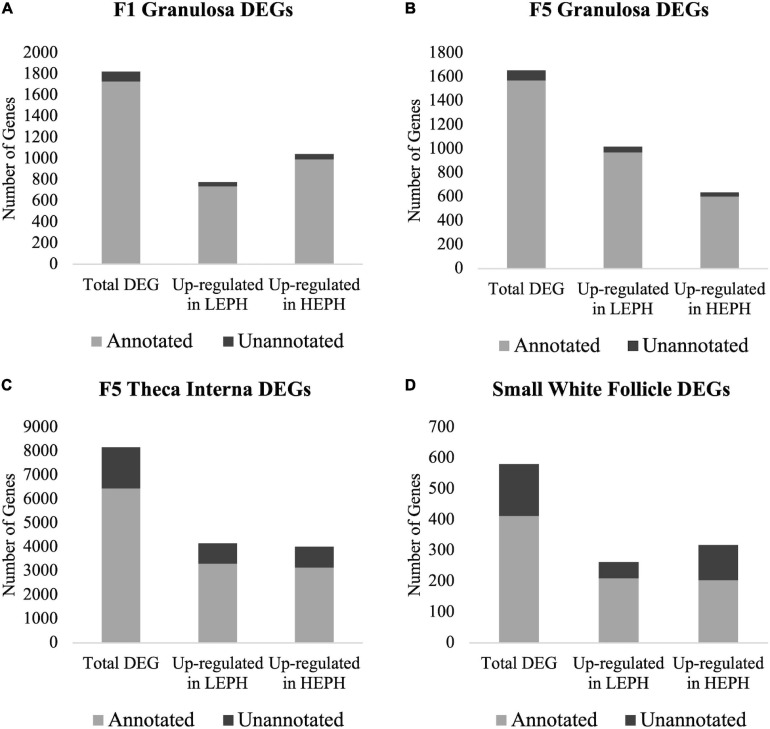
Number of differentially expressed genes. Numbers of total, up-regulated in high egg producing hens (HEPH), and up-regulated in low egg producing hens (LEPH) differentially expressed genes (DEGs) between LEPH and HEPH in **(A)** the F1 granulosa, **(B)** the F5 granulosa, **(C)** the F5 theca interna, and **(D)** the small white follicles (RPKM > 0.2, *P* < 0.05). The portion of genes that are unannotated in the turkey genome are represented in dark gray and the portion of gene that are annotated in the turkey genome are represented in light gray.

A total of 1824, 1654, 8163, and 580 differentially expressed genes (DEGs) between low and high egg producing hens were identified in the F1G, F5G, F5I, and SWF, respectively (Supplementary Additional Files 1–4). The highest number of DEGs between low and high egg producing hens was seen in the F5I, whereas the lowest number of DEGs between low and high egg producing hens was seen in the SWF. In the F1G, a larger percentage of DEGs were up-regulated in high egg producing hens, whereas in the F5G a larger percentage of DEGs were up-regulated in low egg producing hens (Figures 1A,B). In both the F1G and F5G, only a small percentage of genes were unannotated in the turkey genome (5.56 and 5.35%, respectively). In the F5I, roughly equal numbers of the DEGs were up-regulated in low and high egg producing hens (Figure 1C). In the SWF, slightly more DEGs were up-regulated in high egg producing hens compared to those up-regulated in low egg producing hens (Figure 1D). A larger percentage of DEGs in the F5I and SWF were unannotated in the turkey genome compared to the DEGs in the F1G and F5G (26.72 and 40.78%, respectively). A majority of previous studies examining ovarian steroidogenesis and follicle development have focused on the granulosa layer of the follicle wall and may account for the high percentage of unannotated DEGs seen in the F5I and SWF. Additionally, the heterogenous cell populations found in the F5I and SWF, compared to the homogenous cell population found in the granulosa layer, may contribute to the high percentage of unannotated DEGs seen in the F5I and SWF.

**FIGURE 2:**
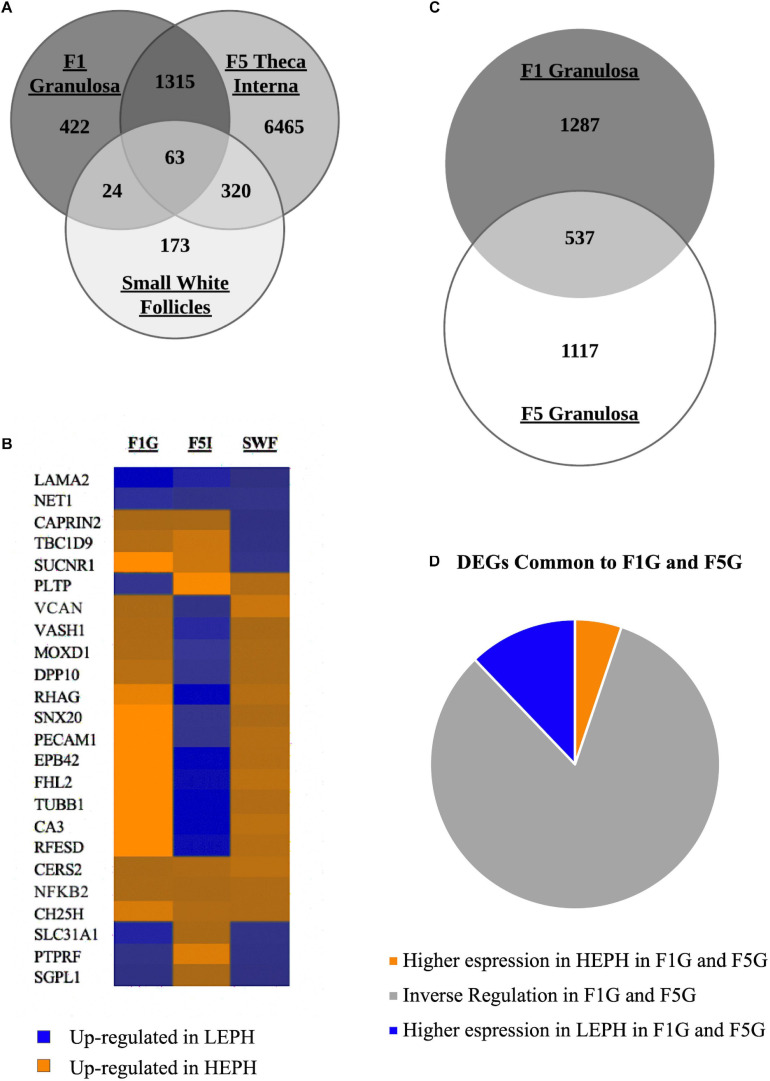
Overview of differentially expressed genes. **(A)** Venn diagram showing the number of differentially expressed genes (DEGs) between low egg producing hens (LEPH) and high egg producing hens (HEPH) that are unique to the F1 granulosa, F5 theca interna, and small white follicles as well as the number of DEGs common to one or more follicle cell types (RPKM > 0.2, *P* < 0.05). **(B)** Heat map showing the expression profiles of the DEGs common to the F1 granulosa, F5 theca interna, and small white follicles displaying high expression in each of the follicle cell types examined (RPKM > 10, *P* < 0.05). Blue represents genes up-regulated in LEPH, whereas orange represents genes up-regulated in HEPH. **(C)** Venn diagram showing the number of DEGs in unique to the F1 granulosa and F5 granulosa as well as the number of DEGs common both the F1 granulosa and F5 granulosa (RPKM > 0.2, *P* < 0.05). **(D)** DEGs common to both the F1 granulosa and F5 granulosa displaying high expression in both follicle cell types broken down by expression pattern in LEPH and HEPH (RPKM > 10, *P* < 0.05).

Of the three cell types primarily responsible for progesterone, androgen, and estradiol production, the F1G, F5I, and SWF, respectively, 422 DEGs were unique to the F1G, 6465 DEGs were unique to the F5I, and 173 DEGs were unique to the SWF (Figure 2A). The F1G and F5I had 1378 DEGs in common, the F5I and SWF had 383 DEGs in common, and the F1G and SWF had 87 DEGs in common. The three cells types had 63 DEGs in common, which displayed differential regulation in low and high egg producing hens across the cell types examined (Figure 2B). The F1G and F5G had 537 genes in common (Figure 2C). A majority of the DEGs between low and high egg producing hens common to both the F1G and F5G showed inverse regulation in the F1G and F5G (83%) (Figure 2D).

### RNA Sequencing Confirmation

**FIGURE 3:**
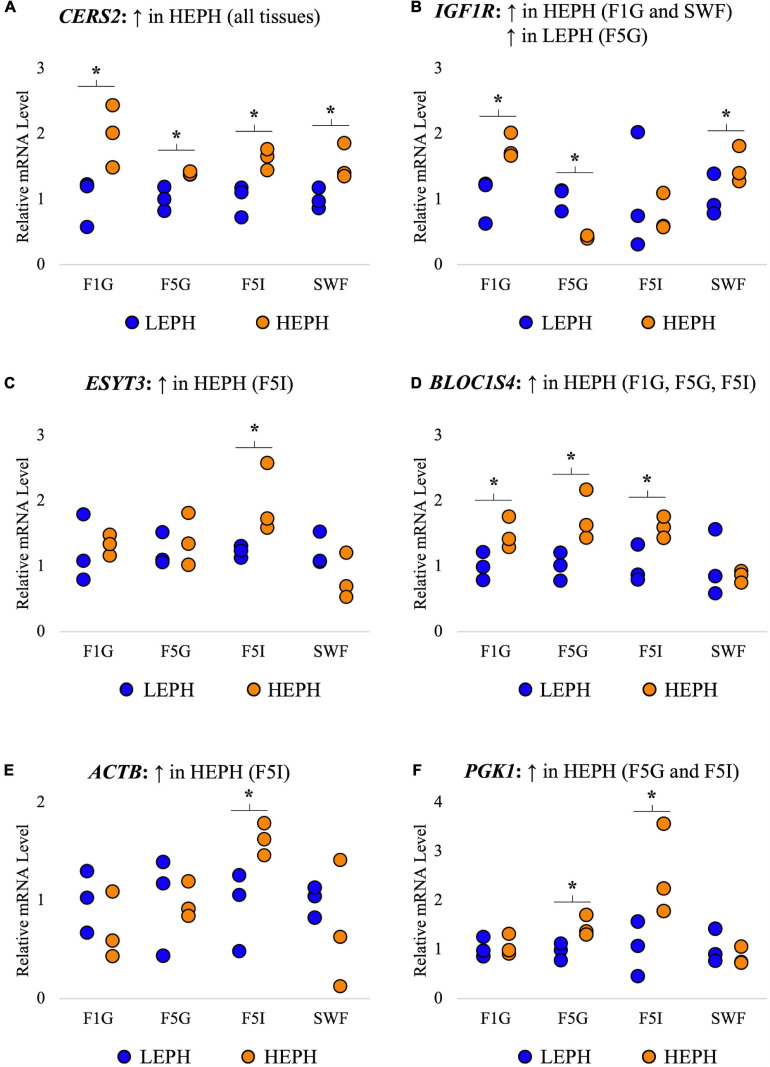
Confirmation of RNAseq gene expression results. Confirmation of RNA sequencing results in the F1 granulosa (F1G), F5 granulosa (F5G), F5 theca interna (F5I), and small white follicles (SWF). A total of 6 genes common to all four tissues that displayed high expression and different expression patterns among the four tissues were selected for confirmation in each tissue through RT-qPCR. The expression differences (*P* < 0.05) between low egg producing hens (LEPH) and high egg producing hens (HEPH) for each gene seen in RNA sequencing results are listed above each graph. Normalized data are presented relative to LEPH expression for each follicle type for each gene. Significant expression differences between LEPH and HEPH for a given follicle type are denoted with an asterisk (*). **(A)** Relative mRNA levels for *ceramide synthase 2* (*CERS2*), which was up-regulated in HEPH in all four tissues in RNA sequencing results. **(B)** Relative mRNA levels for *insulin-like growth factor 1 receptor* (*IGFR1*), which showed increased expression in HEPH in the F1G and SWF and increased expression in LEPH in the F5G in RNA sequencing results. **(C)** Relative mRNA levels for extended *synaptotagmin 3* (*ESYT3*), which was up-regulated in HEPH in the F5I in RNA sequencing results **(D)** Relative mRNA levels for *biogenesis of lysosomal organelles complex 1 subunit 4* (*BLOC1S4*), which showed increased expression in HEPH in the F1G, F5G, and F5I in RNA sequencing results. **(E)** Relative mRNA levels for *beta-actin* (*ACTB*), which showed up-regulation in HEPH in the F5I in RNA sequencing results. **(F)** Relative mRNA levels for *phosphoglycerate kinase 1* (*PGK1*), which showed up-regulation in HEPH in the F5G and F5I in RNA sequencing results.

A total of 6 genes common to all four tissues that displayed high expression levels and different expression patterns among the four tissues were selected for confirmation in each tissue through reverse transcription quantitative PCR (RT-qPCR). RT-qPCR results confirmed increased expression of ceramide synthase 2 (*CERS2*) in high egg producing hens in all four tissues, increased expression of insulin-like growth factor 1 receptor (*IGFR1*) in high egg producing hens in the F1G and SWF as well as in low egg producing hens in the F5G, increased expression of extended synaptotagmin 3 (*ESYT3*) in high egg producing hens in the F5I, increased expression of biogenesis of lysosomal organelles complex 1 subunit 4 (*BLOC1S4*) in high egg producing hens in the F1G, F5G, and F5I, increased expression of beta-actin (*ACTB*) in high egg producing hens in the F5I, and increased expression of phosphoglycerate kinase 1 (*PGK1*) in high egg producing hens in the F5G and F5I (Figure 3). Each of the confirmation genes examined in the F1G, F5G, F5I, and SWF showed expression profiles similar to those obtained through RNA sequencing.

### Network Analysis

**FIGURE 4:**
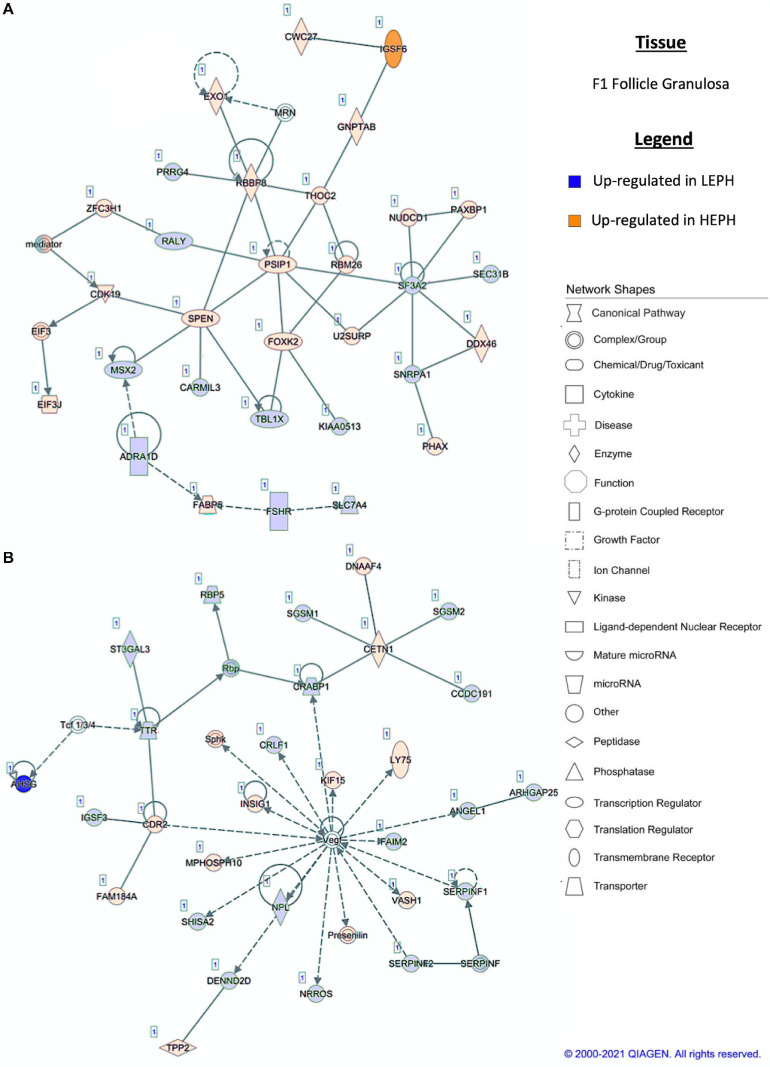
**(A,B)** F1 granulosa pathway analysis. Network analysis in the F1 granulosa comparing low egg producing hen (LEPH) and high egg producing hens (HEPH) gene expression (RPKM > 0.2, *P* < 0.05, | fold change| > 1.5) generated using Ingenuity^®^ Pathway Analysis (Qiagen, Valencia, CA). Blue represents genes up-regulated in LEPH, whereas orange represents genes up-regulated in HEPH. Solid lines represent direct relationships between molecules, whereas dotted lines represent indirect relationships between molecules.

**FIGURE 5:**
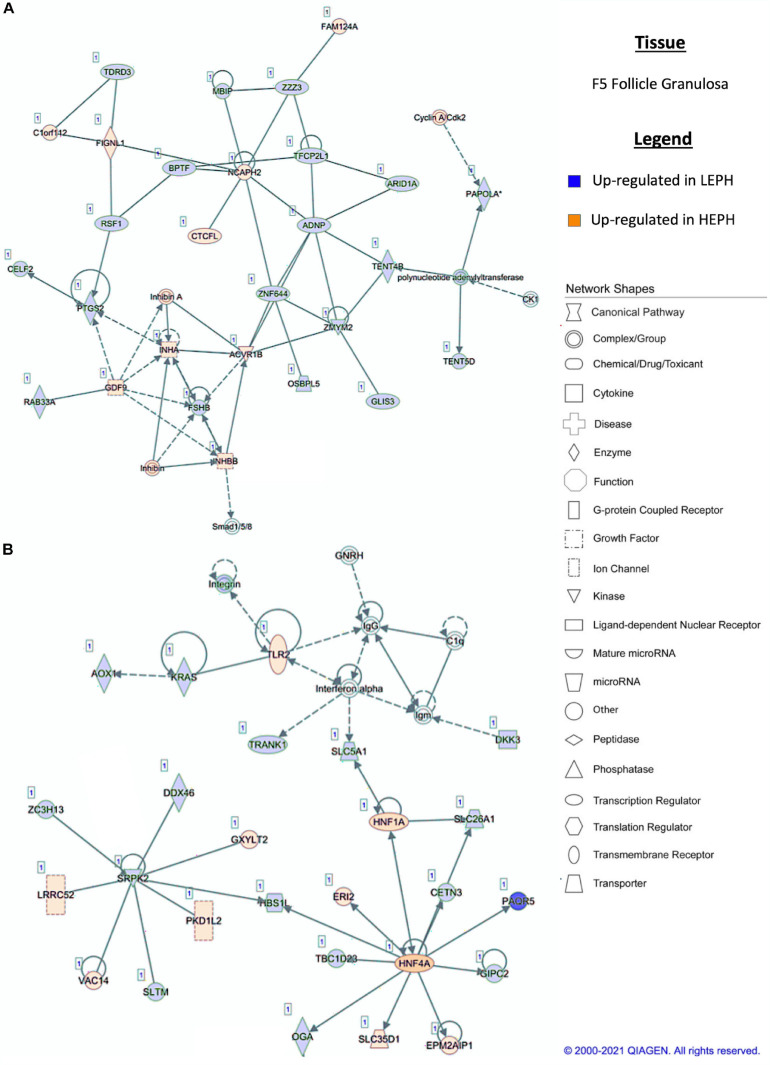
**(A,B)** F5 granulosa pathway analysis. Network analysis in the F5 granulosa comparing low egg producing hens (LEPH) and high egg producing hens (HEPH) gene expression (RPKM > 0.2, *P* < 0.05, | fold change| > 1.5) generated using Ingenuity^®^ Pathway Analysis (Qiagen, Valencia, CA). Blue represents genes up-regulated in LEPH, whereas orange represents genes up-regulated in HEPH. Solid lines represent direct relationships between molecules, whereas dotted lines represent indirect relationships between molecules.

**FIGURE 6:**
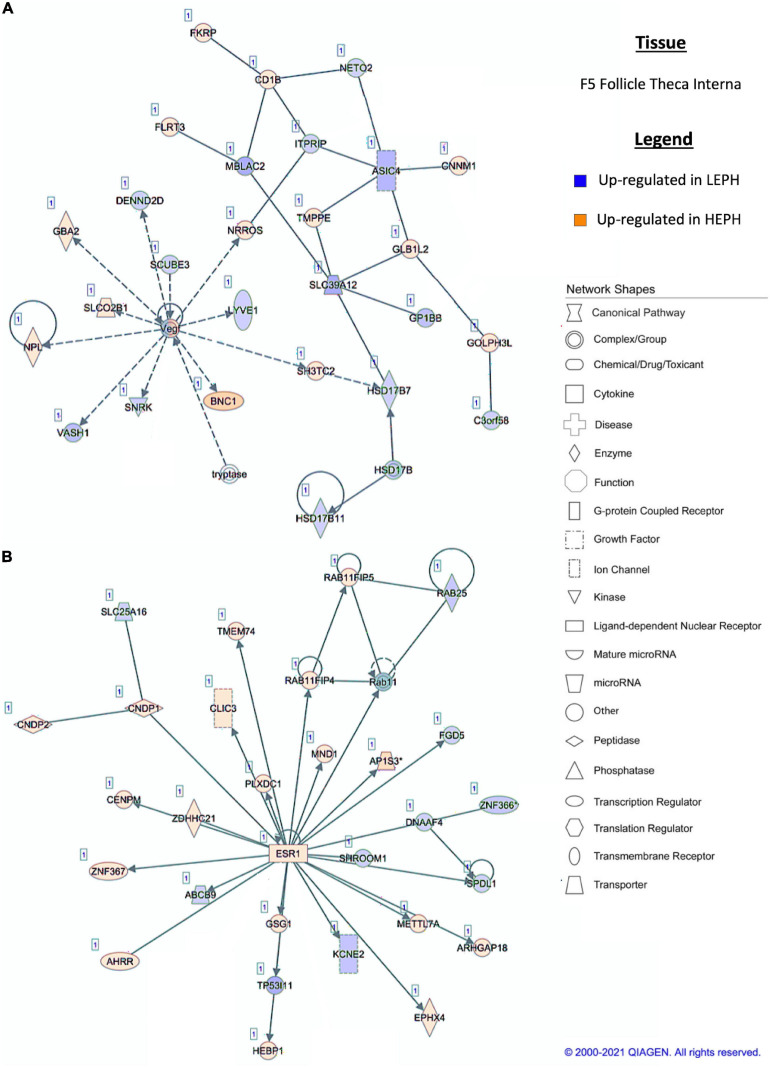
**(A,B)** F5 theca interna pathway analysis. Network analysis in the F5 theca interna comparing low egg producing hens (LEPH) and high egg producing hens (HEPH) gene expression (RPKM > 0.2, *P* < 0.05, | fold change| > 1.5) generated using Ingenuity^®^ Pathway Analysis (Qiagen, Valencia, CA). Blue represents genes up-regulated in LEPH, whereas orange represents genes up-regulated in HEPH. Solid lines represent direct relationships between molecules, whereas dotted lines represent indirect relationships between molecules.

**FIGURE 7:**
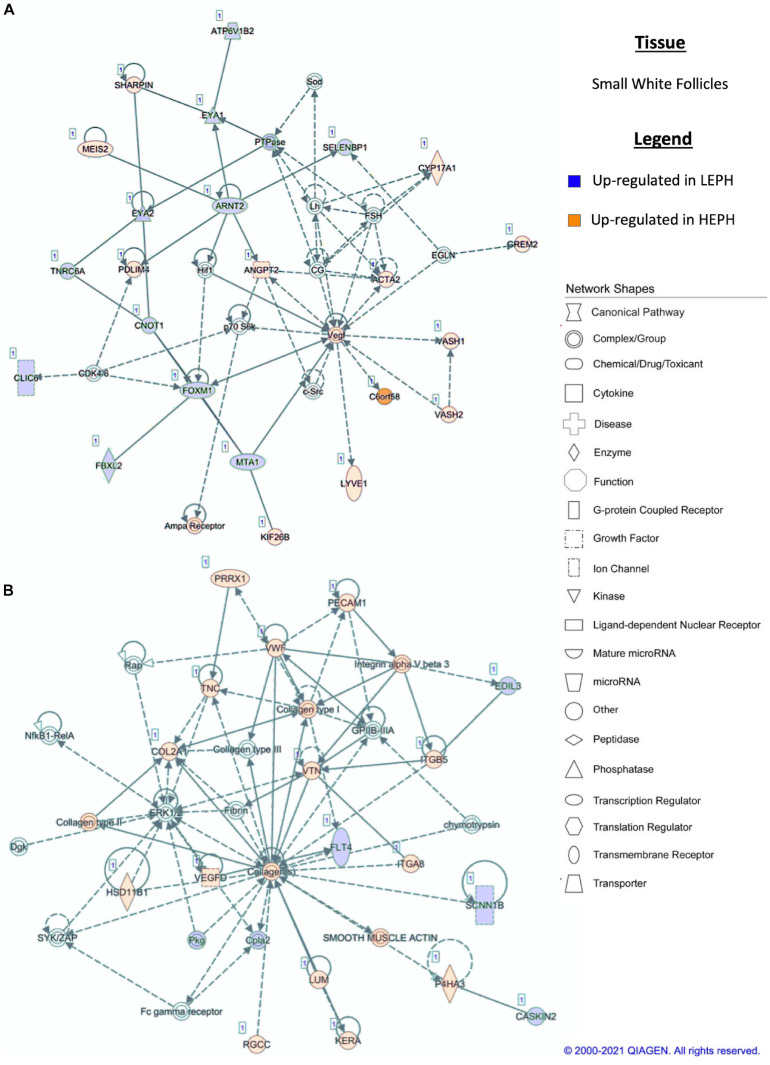
**(A,B)** Small white follicle pathway analysis. Network analysis in the small white follicles comparing low egg producing hens (LEPH) and high egg producing hens (HEPH) gene expression (RPKM > 0.2, *P* < 0.05, | fold change| > 1.5) generated using Ingenuity^®^ Pathway Analysis (Qiagen, Valencia, CA). Blue represents genes up-regulated in LEPH, whereas orange represents genes up-regulated in HEPH. Solid lines represent direct relationships between molecules, whereas dotted lines represent indirect relationships between molecules.

Pathway analysis of the DEGs in F1G exposed aspects seen only in low egg producing hens: gonadotropin receptor up-regulation that is normally observed in less mature follicles (Figure 4A) and thyroid hormone transporter up-regulation (Figure 4B). In the F5G, high egg producing hens displayed increased expression of genes related to inhibin signaling (Figure 5A), whereas, low egg producing hens displayed increased expression of thyroid hormone non-genomic signaling components (Figure 5B). In the F5I, low egg producing hens showed up-regulation androgen production genes (Figure 6A) while high egg producing hens exhibited up-regulation of estrogen signaling genes (Figure 6B). Finally, in the SWF, increased expression of genes involved in estrogen production (Figure 7A) and non-genomic thyroid hormone signaling was seen in high egg producing hens (Figure 7B). Overall, high egg producing hens exhibited increased expression of genes involved in progesterone and estradiol production and low egg producing hens showed increased expression of genes involved in androgen production. Additionally, the HPT axis appeared to be up-regulated in more mature follicles in low egg producing hens and in less mature follicles in high egg producing hens. Lastly, genes related to estradiol signaling showed increased expression in high egg producing hens compared to low egg producing hens.

### Upstream Analysis

Analysis of the predicted upstream regulators for the follicle cell types examined showed a general activation by beta-estradiol. While the calculated z-score varied for the comparisons examined, beta-estradiol was the only upstream regulator common to all of the comparisons. Additionally, beta-estradiol was among the top five upstream regulators in the F1G, F5G, and F5I (Table 1). For the comparisons between low and high egg producing hens, beta-estradiol was significantly more active in high egg producing hens in the F5I (z-score = 2.268) and SWF (z-score = 2.588). While beta-estradiol was a top upstream regulator in the F1G and F5G, the activation z-score was less than 2 in these tissues. Top upstream regulators in the F1G, F5G, F5I, and SWF that showed significant activation z-scores included activation of GATA binding protein 1 (GATA1) (z-score = 2.4) in F1G cells from high egg producing hens, cyclin dependent kinase inhibitor 1A (CDKN1A) (z-score = −2.1) in F5G cells from low egg producing hens, beta-estradiol, tumor necrosis factor (TNF) (z-score = 2.6), and interferon gamma (IFNG) (z-score = 3) in F5I cells from high egg producing hens, and transforming growth factor beta 1 (TGFB1) (z-score = 4.3) and serum response factor (SRF) (z-score = 3.2) in SWF cells from high egg producing hens (Table 1).

**TABLE 1:** Upstream Regulators Top five upstream regulators generated through Ingenuity^®^ Pathway Analysis (Qiagen, Valencia, CA) between low egg producing hens and high egg producing hens for each follicle cell type (RPKM > 0.2, *P* < 0.05, | fold change| > 1.5).

Upstream regulator	Molecule type	Z-score	*P*-value	Target genes
**F1 granulosa**				
TP53	Transcription regulator	0.4	6.4E-18	287
Beta-estradiol	Chemical-endogenous	0.7	6.8E-18	300
Dexamethasone	Chemical drug	0.8	1.7E-17	311
ERBB2	Kinase	–1.8	1.2E-14	140
GATA1	Transcription regulator	2.4	5.7E-14	63
**F5 granulosa**				
ESR1	Ligand-dependent nuclear receptor	–1.0	8.8E-20	204
Beta-estradiol	Chemical-endogenous	0.1	2.7E-19	271
E2F4	Transcription regulator	1.0	2.2E-11	51
CDKN1A	Kinase	–2.1	8.9E-11	49
MITF	Transcription regulator	1.9	4.4E-10	50
**F5 theca interna**				
Beta-estradiol	Chemical-endogenous	2.3	1.4E-20	460
TGFB1	Growth factor	–1.0	1.4E-15	408
TNF	Cytokine	2.6	7.8E-15	403
IFNG	Cytokine	3	6.6E-12	309
Dexamethasone	Chemical drug	0.3	1.1E-11	437
**Small white follicles**				
TGFB1	Growth factor	4.3	7.9E-10	84
SRF	Transcription regulator	3.2	8.5E-10	30
TNF	Cytokine	0.5	1.6E-08	80
SP1	Transcription regulator	0.4	7.8E-08	36
DLL4	Other	1.8	1.5E-07	9

### Effect of Thyroid Hormone on SWF Estradiol Production

**FIGURE 8:**
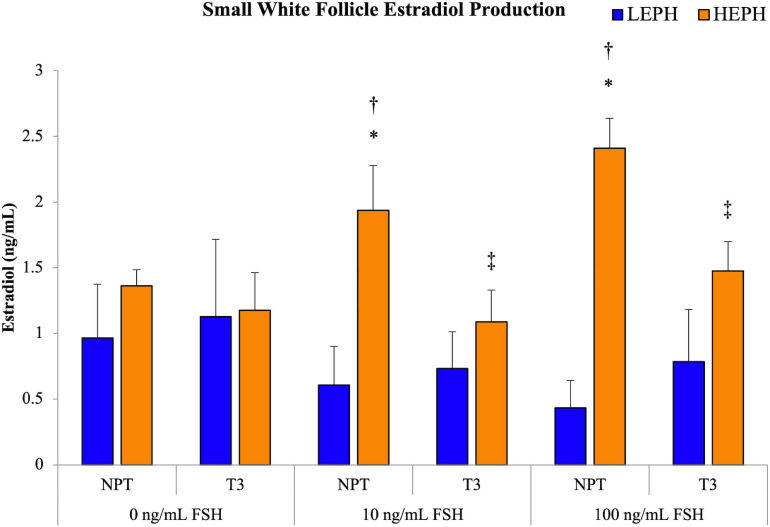
Impact of thyroid hormone on estradiol production. Estradiol production in small white follicle cells from low egg producing hens (LEPH) and high egg producing hens (HEPH) after pretreatment with no pretreatment (NPT) or thyroid hormone (T_3_) followed by treatment with follicle stimulating hormone (FSH). Significant expression differences between LEPH and HEPH for a given condition are denoted with an asterisk (*) (*P* < 0.05). Significant differences between FSH treatments for a given egg production group are denoted with a dagger (†) (*P* < 0.05). Significant differences between pretreatments for a given egg production group are denoted with a double dagger (‡) (*P* < 0.05).

Based on pathway analysis of the follicle cell types, the HPT axis was identified as a possible regulator of the differences in steroidogenesis seen in low and high egg producing hens. To examine if thyroid hormone impacted the elevated estradiol production levels seen previously in high egg producing hens, isolated SWF cells from low and high egg producing hens were subjected to pretreatment with either no pretreatment (NPT) or 1.5 ng/mL of thyroid hormone (T_3_) for 12 h, followed by FSH treatment at 0, 10, and 100 ng/mL for 5 h (Figure 8). Basal estradiol production *in vitro* did not differ between SWF cells from low and high egg producing hens, regardless of pretreatment. However, at 10 and 100 ng/mL treatment of FSH, high egg producing hen SWF cells showed significantly higher estradiol production when compared to cells from low egg producing hens. SWF cells from low egg producing hens did not respond to either FSH treatment in terms of estradiol production. Pretreatment with T_3_, both at 10 and 100 ng/mL of FSH, decreased estradiol production in SWF cells from high egg producing hens, reducing estradiol production to levels seen in low egg producing hens.

## Discussion

Transcriptome analysis of the follicle cell types responsible for progesterone, androgen, and estradiol production in low and high egg producing hens provided insights into the differential regulation of steroidogenesis and follicle development between the two groups of hens. Several trends in gene expression were seen through the comparison of low and high egg producing hens. The first was up-regulation of genes involved in progesterone and estradiol production in high egg producing hens, while low egg producing hens displayed up-regulation of genes involved in androgen production. These results are consistent with previous studies examining low and high egg producing hens (Brady et al., 2020a, b). The second was the up-regulation of HPT axis-related genes in low egg producing hens in more mature follicles, yet the up-regulation of HPT axis-related genes in high egg producing hens in less mature follicles. These results are consistent with the hypothesis that the thyroid axis impacts follicular development and steroidogenesis differently in low and high egg producing hens. This hypothesis was further supported *in vitro*, with thyroid hormone treatment decreasing FSH-stimulated estradiol production in SWF cells from high egg producing hens but not in SWF cells from low egg producing hens. Up-regulation of the HPT axis later in follicle development, as is seen in low egg producing hens, is inconsistent with the marked decreased expression of thyroid hormone receptors during follicle development established in previous studies and indicated that the HPT axis may play a role in slowing the progress of developing follicles through the follicular hierarchy in low egg producing hens. The third was the up-regulation of estradiol signaling in high egg producing hens. Significant activation of beta-estradiol upstream regulation in high egg producing hens was predicted in less mature follicle types.

Generally, across the follicle types examined, low egg producing hens showed expression consistent with a longer progression through the follicular hierarchy. Low egg producing hens displayed up-regulation of genes previously associated with atretic follicles, though macroscopically, atretic follicles were not found in low egg producing hens. These genes may be up-regulated in atretic follicles due to the decrease in follicle cell proliferation and development seen in atretic follicles (Hatzirodos et al., 2014a). Up-regulation of these genes in low egg producing hens throughout follicle development, may imply that follicle cells from low egg producing hens are not developing as fast as those from high egg producing hens. On the other hand, high egg producing hens displayed up-regulation of genes previously associated with healthy follicles, which further supports the hypothesis that follicle development is more rapid in high egg producing hens compared to low egg producing hens. Lastly, low egg producing hens exhibited up-regulation of *FSHR*, which is normally up-regulated earlier in follicle development, whereas high egg producing hens exhibited up-regulation of genes that have been shown to positively regulate the LH receptor gene, which is normally up-regulated at the end of follicle development.

### Overview of DEGs

Of the DEGs between low and high egg producing hens that were common to the F1G, F5I, and SWF, several have been previously identified as coactivators in the regulation of steroidogenesis. For example, knockdown of four and a half LIM domain 2 (*FHL2*) in mice granulosa cells resulted in decreased expression of cholesterol side chain cleavage enzyme (*CYP11A1*), which is involved in progesterone production (Matulis and Mayo, 2012). Additionally, nuclear factor kappa beta-subunit 2 (*NFKB2*) was identified as an upstream regulator active in bovine thecal cells (Hatzirodos et al., 2017). Of the DEGs common to F1G, F5I, and SWF, a majority of the genes displayed inverse expression trends in low and high egg producing hens across the follicle cell types. Higher expression of the common DEGs in high egg producing hens was generally seen in the F1G and SWF, indicating possible roles in the regulation of progesterone and estradiol production. In contrast, higher expression of the common DEGs in low egg producing hens was generally seen in the F5I, indicating possible roles in the regulation of androgen production. Further studies will be necessary to identify possible roles of the common DEGs in steroidogenesis.

In previous studies, low egg producing hens displayed up-regulation of progesterone regulated genes in the F5G, whereas high egg producing hens displayed up-regulation of progesterone regulated genes in the F1G (Brady et al., 2020a). Moreover, progesterone production increases with the amount of time spent in the preovulatory follicle cohort (Johnson and Bridgham, 2001). Comparison of DEGs between low and high egg producing hens in the F1G and F5G, may highlight key differences in movement through the follicular hierarchy. Annexin A2 (*ANXA2*), which plays a role in signal transduction pathways, displayed up-regulation in low egg producing hens in the F1G as well as the F5G and was previously identified as up-regulated in bovine atretic follicles compared to healthy follicles (Hatzirodos et al., 2014a). While atretic follicles where not observed in low egg producing hens, mechanisms similar to those seen in follicle atresia could impact the rate of follicle development in low egg producing hens.

Apolipoprotein A1 (*APOA1*) and clathrin light chain B (*CLTB*) displayed up-regulation in low egg producing hens in the F1G but up-regulation in high egg producing hens in the F5G. *APOA1* encodes the major protein component of high-density lipoprotein and was previously shown to up-regulated in less mature follicles rather than more mature follicles in high egg producing ducks, which is consistent with the expression profile seen in high egg producing hens (Wu et al., 2016). *CLTB* encodes two proteins believed to act as regulatory elements and was previously shown to be down-regulated in the granulosa cells of swine follicles just prior to ovulation, which contrasts with expression seen in low egg producing hens (Bonnet et al., 2008). Additionally, *CLTB* was identified to be up-regulated in preovulatory follicles in low egg producing ducks (Tao et al., 2017). Further investigation into the overlapping DEGs between low and high egg producing hens, especially of those with inverse expression patterns in the F1G and F5G, could provide insight into the possible regulatory mechanisms governing the progesterone production differences seen in low and high egg producing hens.

### Network Analysis

Within the F1G, pathway analysis of DEGs between low and high egg producing hens revealed up-regulation of genes associated with normal follicle function and responsiveness to LH in high egg producing hens as well as up-regulation of genes associated with follicle atresia, thyroid hormone transportation, and responsiveness to FSH in low egg producing hens. The F1G is mainly responsive to stimulation by LH, yet, low egg producing hens displayed up-regulation of follicle stimulating hormone receptor (*FSHR)* when compared to high egg producing hens (Figure 4A). In previous studies, *FSHR* expression was shown to decrease in chicken follicles during maturation (Johnson and Woods, 2009). Up-regulation of *FSHR* may interfere with the actions of LH in the mature follicle. Additionally, high egg producing hens displayed up-regulation of RB binding protein 8 endonuclease (*RBBP8*) and U2 snRNP associated SURP domain containing (*U2SURP*) relative to low egg producing hens, which were also found to be up-regulated in healthy bovine follicles (Hatzirodos et al., 2014b). Low egg producing hens exhibited up-regulation of transthyretin (*TTR*), a thyroid hormone transporter, which could play a role in eliciting the effects of thyroid hormone on the F1G (Figure 4B). Additionally, low egg producing hens displayed increased expression of shisa family member 2 (*SHISA2*), which was also up-regulated in atretic bovine follicles (Hatzirodos et al., 2014a). High egg producing hens showed increased expression of insulin induced gene 1 (*INSIG1*), which has been shown to up-regulate luteinizing hormone receptor (*LHCGR*) in mouse granulosa cells (Menon et al., 2018). *INSIG1* up-regulation of LHCGR in high egg producing hens, coupled with the increased expression of *FSHR* in low egg producing hens, could be responsible for the increased responsiveness of F1G cells from high egg producing hens to LH treatment, while F1G cells from low egg producing hens do not respond to LH treatment (Brady et al., 2020b).

Within the F5G, pathway analysis of DEGs between low and high egg producing hens revealed increased expression of genes related to inhibin signaling and follicle maturation in high egg producing hens and increased expression of genes related to the non-genomic actions of thyroid hormone in low egg producing hens. In the F5G, high egg producing hens exhibited increased expression of inhibin subunit alpha (*INHA*) and inhibin subunit beta B (*INHBB*) compared to low egg producing hens (Figure 5A). Ovarian inhibins feedback on the pituitary as well as exhibit local effects in the ovary related to follicle development (Welt et al., 2002). *INHA* and *INHBB* mRNA levels increase in the granulosa cells during follicle development, suggesting that the F5G from high egg producing hens may be more developed than the F5G from low egg producing hens due to a more rapid follicle development rate to keep up with increased ovulation rates in high egg producing hens (Lovell et al., 1998). Low egg producing hens exhibited increased expression of the integrin receptor in the F5G when compared to high egg producing hens (Figure 5A). The integrin receptor binds thyroid hormones to elicit non-genomic actions of thyroid hormone in target issues. The mRNA levels of the two genes encoding the subunits of the integrin receptor (*ITGAV* and *ITGB3*) decrease significantly with follicle maturation (Sechman, 2012). Up-regulation of the integrin receptor in low egg producing hens is consistent with expression profiles seen in less mature follicles, possibly indicating that follicle from low egg producing hens are moving slower through the follicular hierarchy than in high egg producing hens.

Within the F5I, pathway analysis of DEGs between low and high egg producing hens showed up-regulation of genes related to estrogen signaling and follicle maturation in high egg producing hens and up-regulation of genes related to androgen production and follicle atresia in low egg producing hens. In the F5I, low egg producing hens exhibited increased expression of 17β-hydroxysteroid dehydrogenase 11 (*HSD17B11*), which is involved in androgen synthesis (Figure 6A). This is consistent with previous studies that found genes related to androgen production to be up-regulated in the F5I from low egg producing hens compared to high egg producing hens (Brady et al., 2020a). In addition, low egg producing hens showed higher expression of DENN domain containing 2D (*DENND2D*), which was previously shown to be up-regulated in bovine atretic follicles (Hatzirodos et al., 2014a). High egg producing hens, on the other hand, showed increased expression of estrogen receptor 1 (*ESR1*) and Rho GTPase activating protein 18 (*ARHGAP18*) when compared to low egg producing hens (Figure 6B). Upon estradiol binding, *ESR1* binds estrogen response elements in the promotor region of target genes to regulate transcription (Hammes and Davis, 2015). Up-regulation of *ESR1* could allow high egg producing hens to be more responsive to estradiol acting in a paracrine fashion. *ARHGAP18* modulates cell signaling and previous studies demonstrated that in chicken and bovine follicles, *ARHGAP18* expression increased significantly during follicle development (Hatzirodos et al., 2014b). Up-regulation of *ARHGAP18* in high egg producing hens, once again, supports the hypothesis that follicles from high egg producing hens are moving through the follicular hierarchy quicker than follicles from low egg producing hens. Within the SWF, pathway analysis of DEGs between low and high egg producing hens showed increased expression of genes associated with estradiol production and non-genomic thyroid hormone signaling compared to low egg producing hens. In the SWF, high egg producing hens exhibited up-regulation of 17, 20 lyase (*CYP17A1*), which is involved in androgen and estradiol synthesis (Figure 7A). This supports results from previous studies that found that genes related to estradiol production were up-regulated in SWF in low egg producing hens compared to high egg producing hens (Brady et al., 2020a). High egg producing hens also showed up-regulation of the integrin receptor in SWF relative to levels in SWF from low egg producing hens (Figure 7B). This is in stark contrast to expression pattern of the integrin receptor in the F5G, which showed up-regulation in low egg producing hens compared to high egg producing hens. Previous studies in laying hens have shown that the subunits of integrin receptor display higher expression in SWF compared to more mature follicle types (Sechman, 2012). Up-regulation of the integrin receptor in SWF from high egg producing hens may increase the sensitivity of high egg producing hens to the non-genomic impact of thyroid hormone on steroidogenesis.

### Upstream Analysis

Upstream analysis of all of the tissues examined in this study, indicated activation of beta-estradiol in high egg producing hens compared to low egg producing hens. Beta-estradiol feeds back on the hypothalamus and pituitary to impact the ovulatory process but also exerts local regulation in the ovary (Nelson and Bulun, 2001; Christian and Moenter, 2010). Differentially expressed target genes of beta-estradiol in the F5I and SWF include *CYP17A1*, hydroxysteroid 17 beta dehydrogenase 2 (*HSD17B2*), and aromatase (*CYP19A1*), all of which are involved in androgen and estradiol production. Within the F1G, GATA1 was activated in high egg producing hens and is a transcription factor implicated in cell proliferation as well as in the up-regulation of ovarian expression of hydroxysteroid 3 beta dehydrogenase 1/2 (*HSD3B1/2*), which is one of the enzymes involved in progesterone production (Yu et al., 2019). Differences in mRNA levels for *HSD3B1/2* were not seen in the F1G between low and high egg producing hens in previous studies, though expression levels were not confirmed at the protein level (Brady et al., 2020a). Within the F5G, CDKN1A was significantly activated in low egg producing hens and CDKN1A knockdown has been shown to promote cell proliferation in human granulosa cell lines (Jiang et al., 2015). CDKN1A activation in F5G could slow proliferation of the granulosa layer development in low egg producing hens.

Aside from beta-estradiol, TNF and IFNG were activated in F5I cells from high egg producing hens. TNF has been previously shown to inhibit androgen production *in vitro* in theca interna cells isolated from cattle while IFNG was found to inhibit cell proliferation of the theca interna cells also isolated from cattle (Hatzirodos et al., 2014c; Samir et al., 2017). Genes associated with androgen were found to be up-regulated in low egg producing hens in the F5I in this study and in previous studies, indicating a possible association between increased androgen production and reduced ovulation frequency (Brady et al., 2019, 2020a). In the SWF, TGFB1 and SRF were both activated in high egg producing hens. TGFB1 has been shown to up-regulate FSHR expression in less mature chicken follicles and could play a role in the increased responsiveness of high egg producing hens SWF cells to FSH treatment *in vitro* (Johnson, 2015b; Brady et al., 2020b). SRF is involved in gonadotropin induced expression of early growth response protein 1 (EGR-1) (Hunzicker-Dunn and Maizels, 2006). EGR-1 expression has been linked to up-regulation of *LHCGR* in early rat follicles and in previous studies, high egg producing hens SWF displaying increased *LHCGR* mRNA levels compared to low egg producing hens. Increased expression of the LH receptor in early follicles is important for follicle selection in the preovulatory hierarchy and could indicate that SWF from high egg producing hens are more mature than those found in low egg producing hens (Yoshino et al., 2002; Brady et al., 2020a).

### Effect of Thyroid Hormone on SWF Estradiol Production

Overall, *in vitro*, SWF cells from high egg producing hens were more responsive to T_3_ treatment, with T_3_ treatment significantly decreasing FSH-induced estradiol production. Furthermore, the addition of T_3_ pretreatment caused SWF cells from high egg producing hens to respond to FSH in a similar manner to that seen in cells from low egg producing hens. Based on pathway analysis, thyroid hormone receptors and transporters appeared to be up-regulated low egg producing hens in more mature follicles and up-regulated in high egg producing hens in less mature follicles. Previously, in high egg producing hens, SWF cells were shown to be more responsive to FSH stimulated estradiol production than low egg producing hens and estradiol production levels in cells from low and high egg producing hens from the current study subjected to NPT are consistent with previous results (Brady et al., 2020b). The depression of gonadotropin-stimulated estradiol production following thyroid hormone treatment seen in high egg producing hens is consistent with results previously reported in laying chicken hens (Sechman et al., 2009).

These results suggest that the HPT axis may play a role in differentially regulating estradiol production in the SWF of low and high egg producing hens. Taken together with the up-regulation of integrin subunits in high egg producing hens in SWF network analysis, it could be hypothesized that the impact of thyroid hormone on estradiol production may be elicited through non-genomic mechanisms. Further studies will be necessary to clarify the role of thyroid hormones in the regulation of SWF estradiol production as well as in the regulation of progesterone and androgen production from the F1G and F5I, respectively. Additional studies examining the effect of circulating thyroid hormones on steroid hormone feedback on the hypothalamus and pituitary may explain the impact of thyroid hormone regulation of steroidogenesis on HPG axis function, and ultimately egg production rates.

### Summary

Collectively, based on the transcriptome analysis of the primary steroid hormone producing follicle cell types in low and high egg producing hens, follicle development appears to be more rapid in high egg producing hens compared to low egg producing hens, and may be responsible for the differential steroidogenesis capabilities between low and high egg producing hens. Follicle development differences in low and high egg producing hens may include regulation by thyroid hormone, androgens, and/or estradiol. Aside from differential steroidogenesis, pathway analysis indicates difference between low and high egg producing hens in terms of cell proliferation and apoptosis as well. Further studies will be necessary to clarify the possible role of thyroid hormones and estradiol in follicle development rates in hens with differential egg production.

## Materials and Methods

### Hen Selection and Tissue Collection

All animal procedures were approved by the Institutional Animal Care and Use Committee at BARC and at the University of Maryland (reference numbers 16-002 and XR-16-09). A total of 200 turkey hens from a commercial line (Hendrix Genetics, Kitchener, Ontario) were housed at the Beltsville Agricultural Research Center (BARC) in individual wire cages. Hens were maintained under standard poultry management practices with artificial lighting (14L:10D) and were provided feed *ad libitum* to NRC standards. Daily egg records were used classify hens as low egg producing hens (eggs per day < 0.6) or high egg producing hens (eggs per > 0.8), using cutoffs based on previous studies examining average flock egg production and egg production distribution (Brady et al., 2020a). Sampling occurred at 37 weeks of age and hens were sampled on the second day of the hen’s laying sequence, with a hard-shell egg in the reproduction tract to ensure that sampling occurred during a laying sequence. Hens were euthanized by cervical dislocation prior to sampling of the F1, F5, and SWF.

The timing of the preovulatory surge was predicted using hourly egg records as previously described (Brady et al., 2019). The F1, F5, and SWF were isolated from three low egg producing hens and three high egg producing hens outside of the preovulatory surge (*n* = 3 per group). F1 and F5 follicles were subjected to isolation of the three cell types from the follicle wall and stored at -80°C prior to RNA extraction and sequencing, as described below. Whole SWF samples from low and high egg producing hens were snap frozen in liquid nitrogen and stored at −80°C prior to assessment through RNAseq and RT-qPCR, as described below. Additionally, three low egg producing hens and three high egg producing hens were sampled outside of the preovulatory surge for SWF isolation and culture (*n* = 3 per group). SWF samples from low and high egg producing hens exclusively sampled for cell culture were dispersed prior to culture as described below. Blood samples were taken from all hens prior to sampling, fractionated by centrifugation as previously described, and stored −20°C prior to assessment through a coated tube radioimmunoassay kit (MP Biomedicals, Solon, OH) for plasma progesterone levels as described previously (Brady et al., 2020b). The intraassay coefficients of variation was 2.89% for the progesterone radioimmunoassay.

### Follicle Wall Cell Isolation

The granulosa and theca interna were isolated from the F1 and F5 follicles using an adapted published method, as previously described (Porter et al., 1989; Brady et al., 2019). Briefly, the yolk was drained from each follicle and the follicle was inverted to peel off the granulosa layer. The theca interna layer was scraped from the inverted follicle. All follicle layers were subjected to trypsin dispersion (1 mg/mL) followed by layering onto a Percoll suspension (50%) to remove debris and red blood cells. Only the F1G, F5G, and F5I of the F1 and F5 follicles were used for this study. Isolated cells were snap frozen and stored at −80°C prior to assessment through RNAseq and RT-qPCR, as described below.

### RNA Isolation, cDNA Library Construction, and Sequencing

RNA extraction and quantification from isolated F1G cells, F5G cells, F5I cells, and whole SWF were performed as previously described (Brady et al., 2019). Quantity and quality of the extracted RNA were determined using an Agilent Technologies 2100 Bioanalyzer (Santa Clara, CA, United States) with a high sensitivity RNA chip (RNA integrity number (RIN) values > 9 for all samples). A SMART-Seq Ultra Low Input RNA kit (Takara Bio Inc., Kusatsu, Japan) was used to generate amplified cDNA from 10 ng of starting RNA following the manufacturer’s procedure. An Agencourt AMPure XP kit (Beckman Coulter, Indianapolis, IN) was used to purify amplified cDNA prior to quantification with an Agilent 2100 bioanalyzer and high sensitivity DNA kit (Agilent, Santa Clara, CA). A Nextera XT DNA library kit (Illumina San Diego, CA) was used to generate two sequencing libraries per sample (with unique index pairings) using 150 pg of amplified cDNA and following the manufacturer’s protocol. An Agencourt AMPure XP kit (Beckman Coulter, Indianapolis, IN) was used for library purification prior to quantification with an Agilent 2100 Bioanalyzer and High Sensitivity DNA Kit (Agilent, Santa Clara, CA). Libraries from all 4 tissues were pooled (10 nM) prior to sequencing on an Illumina NextSeq 500 (75 bp paired-end reads) by North Carolina State University’s GSL facility.

### Analysis of Sequencing Data

Sequencing files were deposited to the NIH Short Read Archive (accession numbers SAMN11624512-SAMN11624517, SAMN11624524-SAMN11624529, SAMN11624536-SAMN11624541, and SAMN11624542-SAMN11624547 for F1G, F5G, F5I, and SWF samples, respectively). CLC Genomics Workbench (Qiagen, Valencia, CA) was used for bioinformatic analysis of sequencing data, including read quality assessments as well as read mapping and counting. Low-quality sequences (Phred < 20) were removed prior to mapping. Reads were mapped to the *Meleagris gallopavo* reference genome (Turkey_2.01; ENSEMBL annotation release 98^1^) and total counts were normalized to determine RPKM values using the mapping algorithm from CLC Genomics Workbench. Differential expression was determined using the differential expression for RNA-seq tool from CLC Genomics Workbench, which uses multifactorial statistics based on a negative binomial generalized linear model that does not assume a normal distribution of the error values. For each follicle cell type, a Wald test was used to test for differences between low and high egg producing hens.

Protein sequences for DEGs that were unannotated in the turkey were subjected to orthologous comparisons in human, mouse, and chicken protein sequences using Ensembl BIOMART^2^. If orthologous comparisons exhibited greater than 50% identity at the protein level, gene annotation from the human, mouse, and/or chicken were assumed for DEGs unannotated in the turkey. DEGs were subjected to Ingenuity Pathway Analysis (IPA) (Qiagen, Valencia, CA). Only DEGs with a *P-*value < 0.05, an absolute fold change ≥ 1.5, and an RPKM > 0.2 were used for IPA analysis. As little is currently known about the transcriptomes of the individual cell types of the follicle wall and the turkey genome is not as well annotated as other livestock species, the distribution of log_2_ transformed RPKM values across all comparisons was examined to determine the optimal RPKM cutoff to use during pathway analysis, in order to limit the noise of lowly expressed genes and develop profiles which most accurately reflect the transcriptional status of each cell type. A RPKM cutoff of 0.2 was selected as this value minimized the number of lowly expressed transcripts but did not approach the peak of log2 transformed RPKM expression values for any of cell types examined. Non-transformed RPKM values were used for pathway analysis. Expression networks were generated and scored using the proprietary network generation algorithm from IPA, which uses experimental verified molecular relationships from the Ingenuity Knowledge Database to generate potential molecular interactions based on the expression profiles of the inputted DEGs from a given comparison (Thomas and Bonchev, 2010). Networks with a network score greater than 35 were considered. Potential upstream transcriptional regulators and their associated activation states (presented as a z-score) were predicted based the expression profiles of the DEGs and on the molecular relationships established in Ingenuity Knowledge Database (Krämer et al., 2014). Upstream regulators with an absolute z-score greater than 2 and *P-*value *<* 0.05 were considered to be activated or inhibited, per the manufacturer’s recommendations.

Confirmation of RNAseq results was performed through reverse transcription of extracted RNA followed by RT-qPCR, as previously described (Brady et al., 2019). Data from all follicle cell types were normalized to glyceraldehyde 3-phosphate dehydrogenase (*GAPDH*) using the 2^–ΔΔCt^ method and analyzed as previously described (Brady et al., 2019). *GAPDH* mRNA levels did not differ statistically between egg production groups for each of the tissues examined. For each tissue, mRNA levels for 6 confirmation genes was determined. DEGs selected for RNAseq confirmation fit the following parameters: *P* < 0.05, absolute fold change greater or equal to 1.5, annotated in the turkey genome, encoded by a single transcript, and highly expressed in all follicle cell types examined. Primers were designed as described previously (Brady et al., 2019). Data are presented as fold increase over mRNA levels for low egg producing hens.

### Dispersion, Culture, and Treatment of Small White Follicle Cells

SWF were dispersed, diluted, and cultured as previously described (Brady et al., 2020b). Cells were pretreated with either NPT (10 μl SMEM added) or 1.5 ng/mL of T_3_ for 12 h, followed by treatment with porcine FSH (National Hormone & Peptide Program, Torrance, CA) at 0, 10, or 100 ng/mL for 5 h. Cell maintenance and storage were performed as previously described (Brady et al., 2020b). Media from SWF cultures was assessed for estradiol content through a coated tube radioimmunoassay kit (MP Biomedicals, Solon, OH) as previously described (Brady et al., 2020b). The intraassay coefficients of variation was 4.36% for the estradiol radioimmunoassay.

### Statistics

Data were analyzed using SAS software (SAS Institute, Cary, NC). Normalized RT-qPCR data for RNA sequencing confirmation genes were log_2_ transformed prior to statistical analysis. A two-way ANOVA using the mixed models procedure (PROC MIXED) was used to analyze mRNA levels of RNAseq confirmation genes from the F1G, F5G, F5I, and SWF samples. A three-way ANOVA using the mixed models procedure (PROC MIXED) was used to analyze estradiol production from SWF cell culture samples subjected to different pretreatment and treatment combinations. The least squares means were compared using the test of least significant difference (PDIFF statement).

## Data Availability Statement

The FASTQ sequencing file datasets supporting the results of this article are available in the NCBI Short Read Archive (SRA; https://www.ncbi.nlm.nih.gov/sra), accession numbers, SAMN11624512-SAMN11624517, SAMN11624524-SAMN11624529, SAMN11624536-SAMN11624541, and SAMN11624542-SAMN11624547 for F1G, F5G, F5I, and SWF samples, respectively. Differential expression output generated or analyzed during this study for each cell type are included in this published article (Additional Files 1–4).

## Ethics Statement

The animal study was reviewed and approved by the Institutional Animal Care and Use Committee at BARC and at the University of Maryland (reference numbers 16-002 and XR-16-09).

## Author Contributions

TP, KB, and JL conceived and designed the experiments. TP and KB collected the samples. KB isolated the RNA and analyzed the results. H-CL and JH constructed the libraries and performed the sequencing. All authors contributed to writing the manuscript.

## Conflict of Interest

The authors declare that the research was conducted in the absence of any commercial or financial relationships that could be construed as a potential conflict of interest.

## Footnotes

1 https://uswest.ensembl.org/Meleagris_gallopavo/Info/Index

2 https://useast.ensembl.org/info/data/biomart/index.html

## Supplementary Material

Supplementary Figure 1 Transcriptome alignment and mapping. **(A)** The number of reads obtained for each sample. The portion of mapped reads for each sample is represented in light gray, whereas the portion of unmapped reads for each sample is represented in dark gray. **(B)** The number of aligned pairs obtained for each sample. The portion of aligned pairs with proper alignment is represented in light gray, with discordant alignment in yellow, and with multiple alignments in dark gray.

Supplementary Additional File 1 Granulosa of the largest follicle (F1G) differential expression between low egg producing hens and high egg producing hens.

Supplementary Additional File 2 Granulosa of the fifth largest follicle (F5G) differential expression between low egg producing hens and high egg producing hens.

Supplementary Additional File 3 Theca interna of the fifth largest follicle (F5I) differential expression between low egg producing hens and high egg producing hens.

Supplementary Additional File 4 Small white follicle (SWF) differential expression between low egg producing hens and high egg producing hens.
