# Supplementary figures and images for: Transcriptome Analysis During Follicle Development in Turkey Hens With Low and High Egg Production
Source: Front Genet. 2021 Mar 18;12:619196. doi: 10.3389/fgene.2021.619196 (PMC8012691; doi:10.3389/fgene.2021.619196)

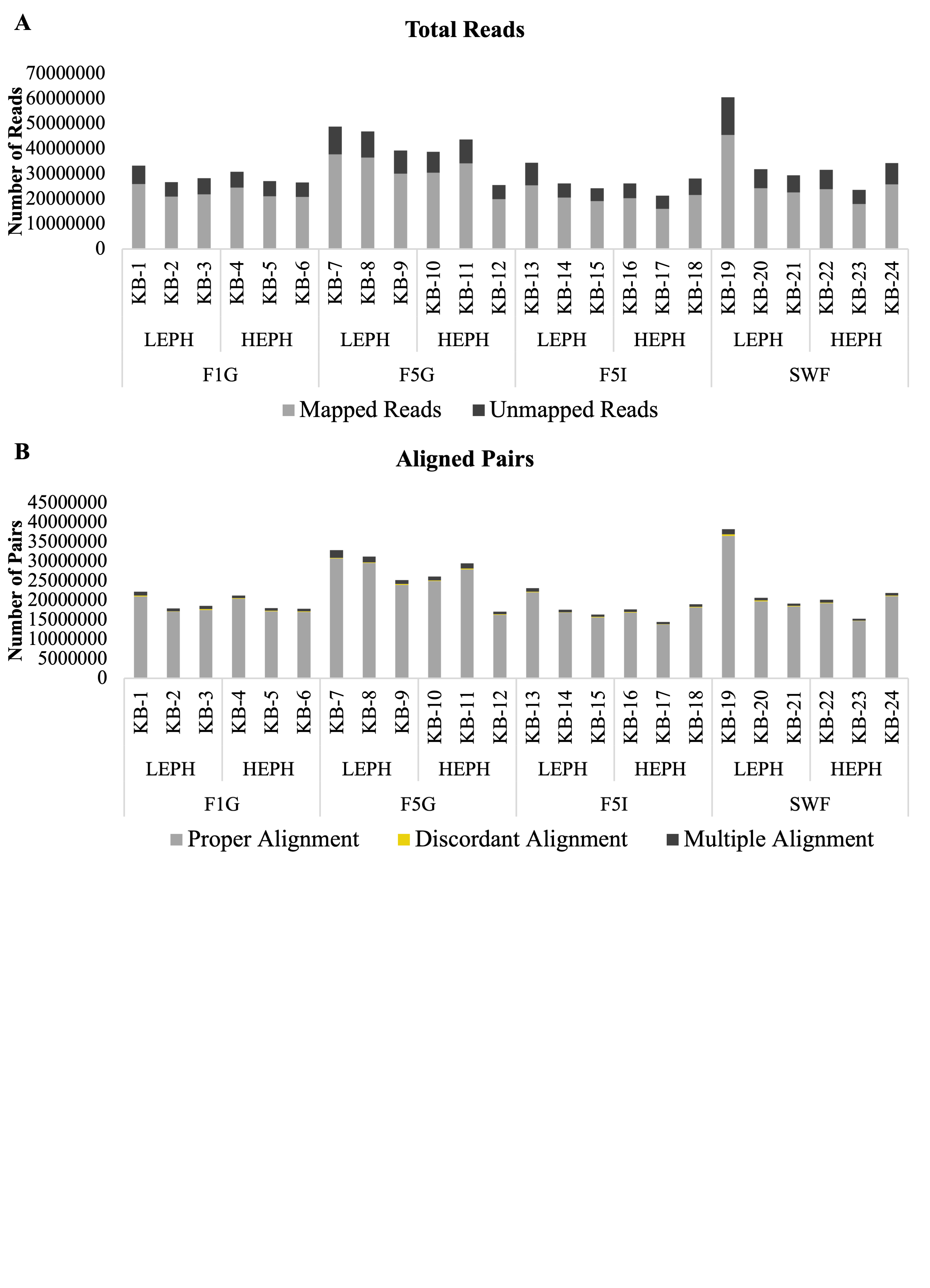

Supplement: Supplementary Figure 1 — Transcriptome alignment and mapping. (A) The number of reads obtained for each sample. The portion of mapped reads for each sample is represented in light gray, whereas the portion of unmapped reads for each sample is represented in dark gray. (B) The number of aligned pairs obtained for each sample. The portion of aligned pairs with proper alignment is represented in light gray, with discordant alignment in yellow, and with multiple alignments in dark gray. [file Image_1.png]
